# Corrigendum to: The DNA intercalators ethidium bromide and propidium iodide also bind to core histones

**DOI:** 10.1002/2211-5463.13397

**Published:** 2022-04-05

Amrita Banerjee, Parijat Majumder, Sulagna Sanyal, Jasdeep Singh, Kuladip Jana, Chandrima Das, Dipak Dasgupta

The original article [1] contained mistakes in the western blots presented in Fig. 3A and B. The corrected version of Fig. 3 is shown below. The conclusions were unaffected by the mistakes and are still supported by the corrected data.

**Fig. 3:**
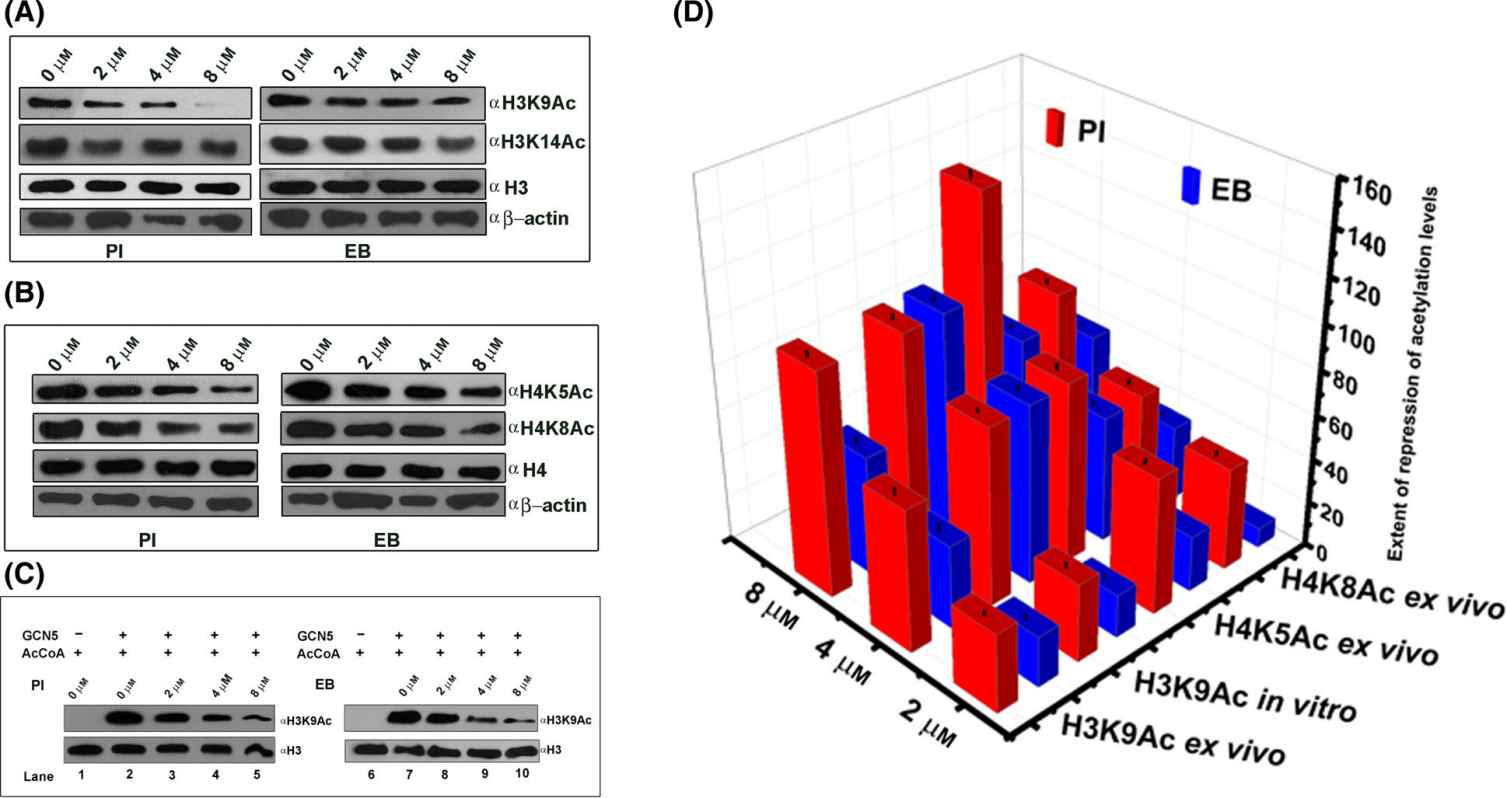
Modulation of acetylation levels of histones H3 and H4 by the ligands (PI and EB) monitored by western blot analyses: (A) Effect of PI/EB on histone H3 acetylation at lysine 9 and 14 in HeLa cells. β‐actin and histone H3 have been used as loading controls. (B) Ligand‐induced alteration of histone H4 acetylation at lysine 5 and 8 in HeLa cells. β‐actin and histone H4 have been used as loading controls. (C) Histone acetyltransferase (HAT) assay performed in the absence and the presence of the ligands using purified HeLa core histones (2 μg) and processed for immunoblot analysis. No enzyme (lane 1 and 6) and no ligand (lanes 2 and 7) controls are shown. Alteration of histone H3K9Ac status in the presence of increasing concentrations (2, 4, and 8 μm) of PI and EB is shown in lanes 3–5 and lanes 8–10, respectively. Loading and transfer of equal amounts of core histones were confirmed by immunodetection of histone H3. (D) Quantification of the extent of ligand‐induced hypoacetylations. The extent of repression of acetylation marks (H3K9Ac, H4K5Ac, and H4K8Ac) in the presence of the ligands has been quantified using image j software and represented in the 3D plot against different concentrations of the ligands. The autoradiograms were quantified using image j software after normalizing with respective histone H3 and H4 loading controls. Then, the fold change was calculated in origin. The error bars were estimated from three independent sets of experiments.
